# Epidemiology, pathology, prevention, and control strategies of inclusion body hepatitis and hepatitis-hydropericardium syndrome in poultry: A comprehensive review

**DOI:** 10.3389/fvets.2022.963199

**Published:** 2022-10-11

**Authors:** Nahed A. El-Shall, Hatem S. Abd El-Hamid, Magdy F. Elkady, Hany F. Ellakany, Ahmed R. Elbestawy, Ahmed R. Gado, Amr M. Geneedy, Mohamed E. Hasan, Mariusz Jaremko, Samy Selim, Khaled A. El-Tarabily, Mohamed E. Abd El-Hack

**Affiliations:** ^1^Poultry and Fish Diseases Department, Faculty of Veterinary Medicine, Alexandria University, Alexandria, Egypt; ^2^Poultry and Fish Diseases Department, Faculty of Veterinary Medicine, Damanhour University, Damanhour, Egypt; ^3^Poultry Disease Department, Faculty of Veterinary Medicine, Beni-Suef University, Beni-Suef, Egypt; ^4^Bioinformatic Department, Genetic Engineering and Biotechnology Research Institute, University of Sadat City, El Sadat City, Egypt; ^5^Smart-Health Initiative and Red Sea Research Center, Division of Biological and Environmental Sciences and Engineering, King Abdullah University of Science and Technology, Thuwal, Saudi Arabia; ^6^Department of Clinical Laboratory Sciences, College of Applied Medical Sciences, Jouf University, Sakaka, Saudi Arabia; ^7^Department of Biology, College of Science, United Arab Emirates University, Al-Ain, United Arab Emirates; ^8^Khalifa Center for Genetic Engineering and Biotechnology, United Arab Emirates University, Al-Ain, United Arab Emirates; ^9^Harry Butler Institute, Murdoch University, Murdoch, WA, Australia; ^10^Poultry Department, Faculty of Agriculture, Zagazig University, Zagazig, Egypt

**Keywords:** aviadenovirus, diagnosis, disease transmission, epidemics, fowl adenoviruses, poultry diseases, vaccines

## Abstract

Infection with fowl adenoviruses (FAdVs) can result in a number of syndromes in the production of chicken, including inclusion body hepatitis (IBH), hepatitis-hydropericardium syndrome (HHS), and others, causing enormous economic losses around the globe. FAdVs are divided into 12 serotypes and five species (A–E; 1–8a and 8b−11). Most avian species are prone to infection due to the widespread distribution of FAdV strains. The genus aviadenovirus, which is a member of the adenoviridae family, is responsible for both IBH and HHS. The most popular types of transmission are mechanical, vertical, and horizontal. Hepatitis with basophilic intranuclear inclusion bodies distinguishes IBH, but the buildup of translucent or straw-colored fluid in the pericardial sac distinguishes HHS. IBH and HHS require a confirmatory diagnosis because their clinical symptoms and postmortem abnormalities are not unique to those conditions. Under a microscope, the presence of particular lesions and inclusion bodies may provide clues. Traditional virus isolation in avian tissue culture is more delicate than in avian embryonated eggs. Additionally, aviadenovirus may now be quickly and precisely detected using molecular diagnostic tools. Preventive techniques should rely on efficient biosecurity controls and immunize breeders prior to production in order to protect progeny. This current review gives a general overview of the current local and global scenario of IBH, and HHS brought on by FAdVs and covers both their issues and preventative vaccination methods.

## Introduction

Avian adenoviruses of the family adenoviridae are found all over the world, infecting a wide variety of poultry hosts and ages, including falcons, raptors, ostriches, psittacine, and parrots (1). Chickens are the primary host for some avian adenovirus diseases such as inclusion body hepatitis (IBH), hepatitis-hydropericardium syndrome (HHS), adenoviral gizzard erosion (AGE), avian adenoviral splenomegaly (AAS), and egg drop syndrome (EDS). All of these diseases are caused by different virus types belonging to different adenovirus genera (Table 1). Recently, IBH and HHS have been widely distributed in broiler flocks in several countries (2–4), and there has been a trend toward more epidemic breakouts rather than sporadic epidemics. Furthermore, the significant mortality and growth retardation associated with IBH, and HHS have resulted in enormous economic losses (2–4).

**Table 1:** Classification of adenoviruses affecting poultry [modified from (1)].

**Family**	**Genus**	**Species**	**Serotype**	**Host range**	**Disease**
Adenoviridae	Aviadenovirus	Fowl adenovirus	A	1	Chicken, quail, guinea fowl, ostrich	CELO, AGE, QB
			B	5	Chicken, pigeon	
			C	4, 10	Chicken, psittacine	HHS
			D	2, 3, 9, 11	Chicken, pigeon, ostrich	IBH
			E	6, 7, 8a, 8b	Chicken, ostrich, pigeon	
		Duck adenovirus	B	2	Muscovy duck	
		Turkey adenovirus	B	1, 2	Turkey	HE, MSD, AAS
			C (2)	4		
			D (2)	5		
		Goose adenovirus	A	1	Goose	
		Falcon adenovirus	A	1	Aplomado/orange-breasted/teita falcon, kestrel	
		Pigeon adenovirus	A	1	Pigeon	
			B (3)	2A, 2B		
	Siadenovirus	Turkey adenovirus	A	3	Turkey	HE, MSD, AAS
		Raptor adenovirus	A	1	Raptor	
	Atadenovirus	Duck adenovirus	A	1	Duck	EDS76

CELO, Chicken embryo lethal orphan; QB, quail bronchitis; IBH, inclusion body hepatitis; HHS, hepatitis-hydropericardium syndrome; AGE, adenoviral gizzard erosion; AAS, avian adenoviral splenomegaly; EDS, egg drop syndrome; MSD, marble spleen disease; HE, hemorrhagic enteritis.

In Egypt, the poultry industry, particularly the broiler chicken sector, is currently experiencing high losses as a result of a high mortality rate, a poor feed conversion ratio, and low weight gain. By diagnosis, it was revealed that they are mostly viral outbreaks such as avian influenza (AI-H5Nx and H9N2), Newcastle disease (ND), infectious bronchitis, and infectious bursal disease (IBD) (5–8), despite extensive vaccination. Vaccination failure may be one of a variety of factors contributing to these losses and another common factor is the immune suppression. As a result, the question here is whether the adenovirus infection is to be blamed for vaccination failure because of its immune suppressive effect (indirect effect) or is it possible that the adenovirus itself is to be blamed for the death and economic losses (direct effect).

The purpose of this current review is to provide an overview of the current global and local situation of IBH, and HHS caused by fowl adenoviruses (FAdVs) and explains their problems and vaccination strategies, in addition to underly the desired procedures that are urgently required to investigate FAdV infections and how to control the losses in poultry industry.

## A brief history

**Figure 1:**
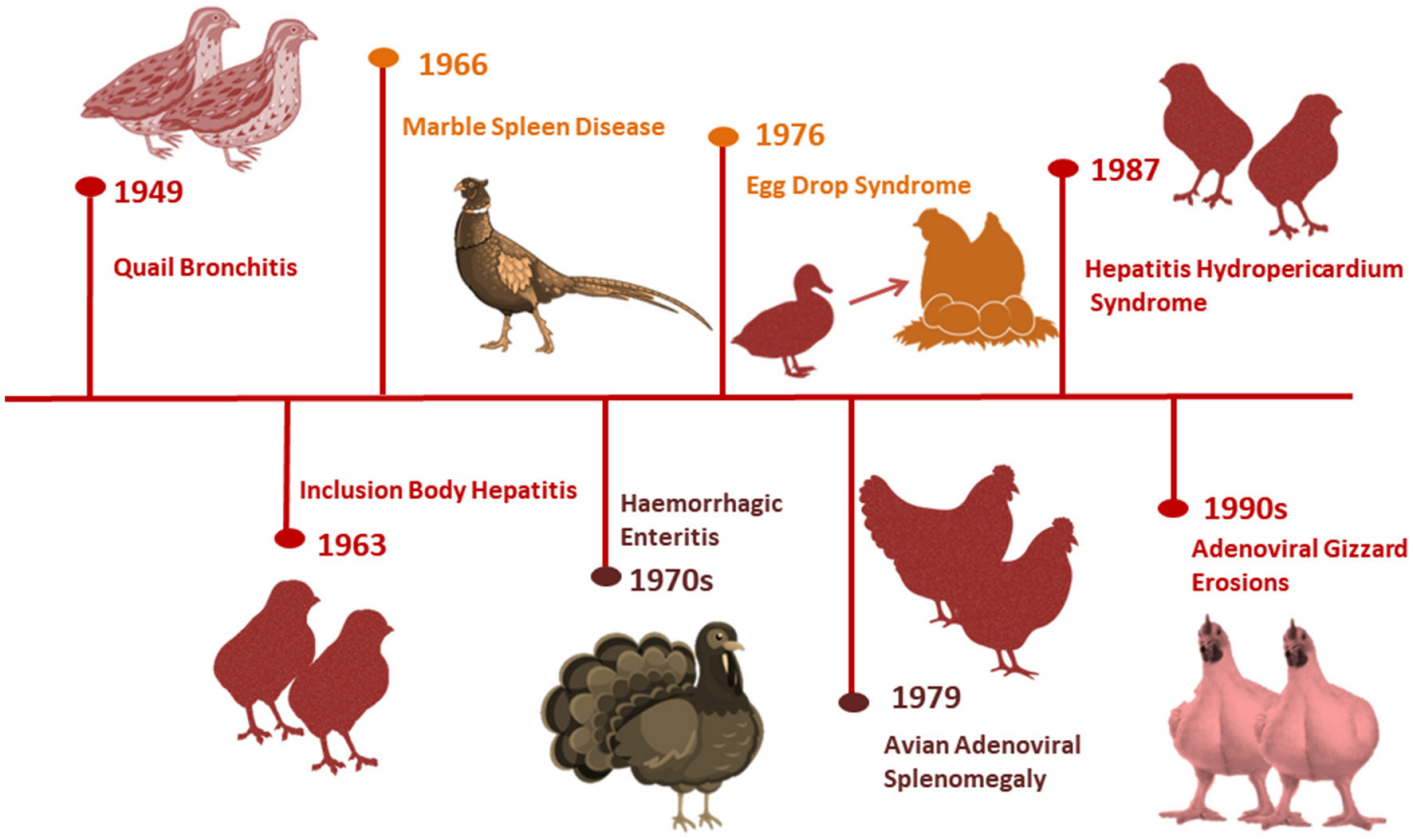
History of avian adenoviruses-associated diseases emergence all over the world.

Figure 1 summarizes the history of adenovirus emergence and identification in poultry. The first aviadenovirus was discovered by chance in the 1950's from embryonated chicken eggs used for diagnosing or preparing vaccines for other non-avian pathogens (9). It was later isolated from Bobwhite quail in West Virginia, USA suffering from respiratory manifestations (10) and named quail bronchitis (QB) disease (11).

Adenoviruses were found in both healthy and diseased birds, and they were all characterized by being replicated in the host cell nucleus and vertically transmitted (11). Several adenovirus-associated outbreaks have emerged in the years since IBH was first detected in two broiler chicken flocks with high mortalities in the USA in 1963 (12). Marble spleen disease (MSD) infiltrated the lungs and spleens of ring-necked pheasants in 1966 (13). Hemorrhagic enteritis (HE), a turkey disease, was discovered to be caused by an adenovirus in the mid-1970's (14), despite outbreaks occurring since the 1930's (15). EDS outbreaks were first reported in laying hens with a soft shell and shell-less egg in the Netherlands in 1976 (16), and were later thought to be initiated from ducks as a natural host *via* contaminated vaccines (17).

AAS was described nearly 3 years later in broiler breeder chickens with enlarged spleens and pulmonary edema (18), but the disease was sporadic and subclinical. Sudden and high mortality in broiler chickens aged 3–6 weeks were first reported in Pakistan in 1987 (Angara Disease; HHS) (19). Tanimura et al. (20) discovered AGE associated with adenovirus intranuclear inclusion bodies (INIB) in layer chickens in Asia, but likes HE, it has been present since the 1930's. IBH and HHS outbreaks brought on by various FAdV serotypes have been reported globally in recent years. With serotypes 2, 8a, 8b, 11, and 4 being the most frequently involved, and the genotypes FAdV-D, -E, or -C have been related to the bulk of these outbreaks (4, 21).

Due to the variety in illness associations, it is currently difficult to determine the overall economic significance of aviadenoviruses (1). However, the high mortality observed in flocks with HHS or IBH, as well as the growth limitation caused by IBH and AGE, result in significant economic losses (4). In rare cases, IBH has been reported in layers and broiler breeders in addition to broilers. Mortality peaks during IBH outbreaks occurs in 3–4 days and can reach 10%, and on rare occasions, 30% (4), especially when concomitant infections are present. This may help to explain why some elements of field outbreaks are difficult to replicate in an experiment.

HHS is clinically similar to IBH, but the mortality in HHS may reach 80% (4, 22, 23) and is characterized by poor development, apathy, prostration, ruffled feathers, and huddling behavior which are some of the clinical indications that may be observed in affected chickens (4, 24). In addition, Bertran et al. (25) mentioned that the the dramatic increase in IBH cases caused major economic losses in the Spanish poultry industry, which prompted the use of vaccination in broiler breeders.

## Current status of IBH and/or HHS associated problems

In recent years, regional and global attention has been focused on the occurrence and economic significance of IBH and HHS and there is an increase in the number of IBH and/or HHS epidemics caused by FAdV over the past 20 years. For instance, FAdV-4 was the dominant serotype in China. Chen et al. (21) isolated 155 FAdV strains from diseased chickens during 3 years (2015–2018) from which FAdV-4 was the dominant serotype representing 79.4% (123/155), while FAdV-8a and FAdV-8b were identified by 13.5 and 3.9%, respectively through the phylogenetic analysis of hexon sequences (21).

In another study by Cui et al. (26) conducted also in China between 2015 and 2018, FAdV-4 was identified in 48 out of 73 hexon-sequencing isolates, whereas 24 of them belonged to serotype 10 (FAdV-10), and one to serotype 2 (FAdV-2). In the USA, IBH was isolated from 15 broiler flocks of age 18–35 days suffering from classic hepatic pathology and swollen kidney (27). Mete et al. (28) reported FAdV4 for the first time in California in a mixed chicken of different breeds and ages (6 months to 2 years old) with a mortality rate of 27% and hydropericardium. In Canada, Philippe et al. (29) isolated serotype 2 from 10-day old broiler breeder pullets experienced a sudden increase in mortality (2.1%). FAdV 8b serotype has been isolated as well by Dar et al. (30) from liver of a 17-day-old broiler chicken flock.

In addition, several reports of IBH and/or HHS were documented in Japan (31), Korea (32), India (33), Pakistan (34), Spain (25), Poland (35), Iran (36), Mexico (37), Brazil (38), Croatia (39), Australia (40), Greece (41), Iraq (42), Palastine (43), United Arab Emirates (44), Saudi Arabia (45), Morocco (46), and South Africa (47).

In Egypt, Mousa et al. (48) detected IBH during the late last century, then El-Shamy (49) detected the IBH histopathologically in the liver and pancreas samples of three broiler chicken flocks suffering from high morbidity (28%) and low mortality rate (2%). There were no subsequent records of its isolation or detection, either experimentally or in the field, until 2015. The detection of IBH (FAdV-E serotype 8a) in 2015 was from two out of nine surveyed (2/9) commercial broiler flocks aged 35 days in El-Beheira province, Egypt by Radwan et al. (50). The commercial flocks had mortality rates ranging from 7 to 17%, a European performance index of 143–212, as well as observable ascites and an enlarged fibrotic liver. However, when the pathogenicity of the isolated and molecularly identified virus was tested in 4-day-old specific-pathogen-free (SPF) layer chicks *via* oral and nasopharyngeal inoculation with 10^4^ tissue culture infective dose_50_ ml^−1^ (TCID_50_ ml^−1^), only severe hepatitis and INIB were observed without mortality (50).

El-Tholoth and Abou El-Azm (51) conducted a case-control study in Kafr EL-Sheikh province, Egypt in the Spring of 2017 to assess IBH incidence. The flocks under investigation were 10 IBH suspected Ross and Sasso broiler chicken flocks aged 7–21 days with a mortality rate of 2.7–15% in comparison to an equal number of control flocks clinically appearing normal. FAdV was examined by polymerase chain reaction (PCR) in 100 liver and spleen samples (10 pooled samples flock^−1^), as well as IBD and chicken infectious anemia virus (CIAV) by PCR for bursa of Fabricius and ELISA, respectively. Not only was FAdV-D detected in seven of 10 flocks examined, but CIAV antibodies were also detected in all IBH positive flocks, despite the absence of IBD infection. Unfortunately, the CIAV status in Egyptian poultry flocks is unknown, so the alarm should sound not only for IBH, but also for CIAV, either as a single or co-infection (51).

Elbestawy et al. (52) detected and characterized FAdV species D (serotype 2 and 11) in 17 out of 37 broiler chicken flocks in northern Egypt during 2017–2018 (Kafr El-Sheikh, Beheira, and Alexandria governorate, Egypt). Sixteen positive flocks died at rates ranging from 0.4 to 16.6%, with one flock dying at a rate of 20% due to co-infection with a highly virulent IBD virus. Furthermore, ecchymotic hemorrhages on enlarged friable livers with hydropericardium were observed, as well as histopathological INIB in hepatocytes (52).

El-Basrey et al. (53) detected IBH in the livers of three out of 40 samples (7.5%) broiler flocks aged 3–5 weeks in Sharkia governorate, Egypt, based on hexon loop-1 gene detection using PCR. The only observed signs with varying mortality were depression and ruffled feathers (8–14%). The three positive samples (EG101/2018, EG102/2018, and EG103/2018) had nucleotide sequence identities of >97.7% and were genetically more similar to reference strains serotype 2 (FAdV-2). Two isolates out of 15 samples from broilers aged 19–40 days suffering from deaths of 5–10% were positive to FAdV-E 8a in Sharkia governorate, Egypt in the period 2019–2020 (54).

**Figure 2:**
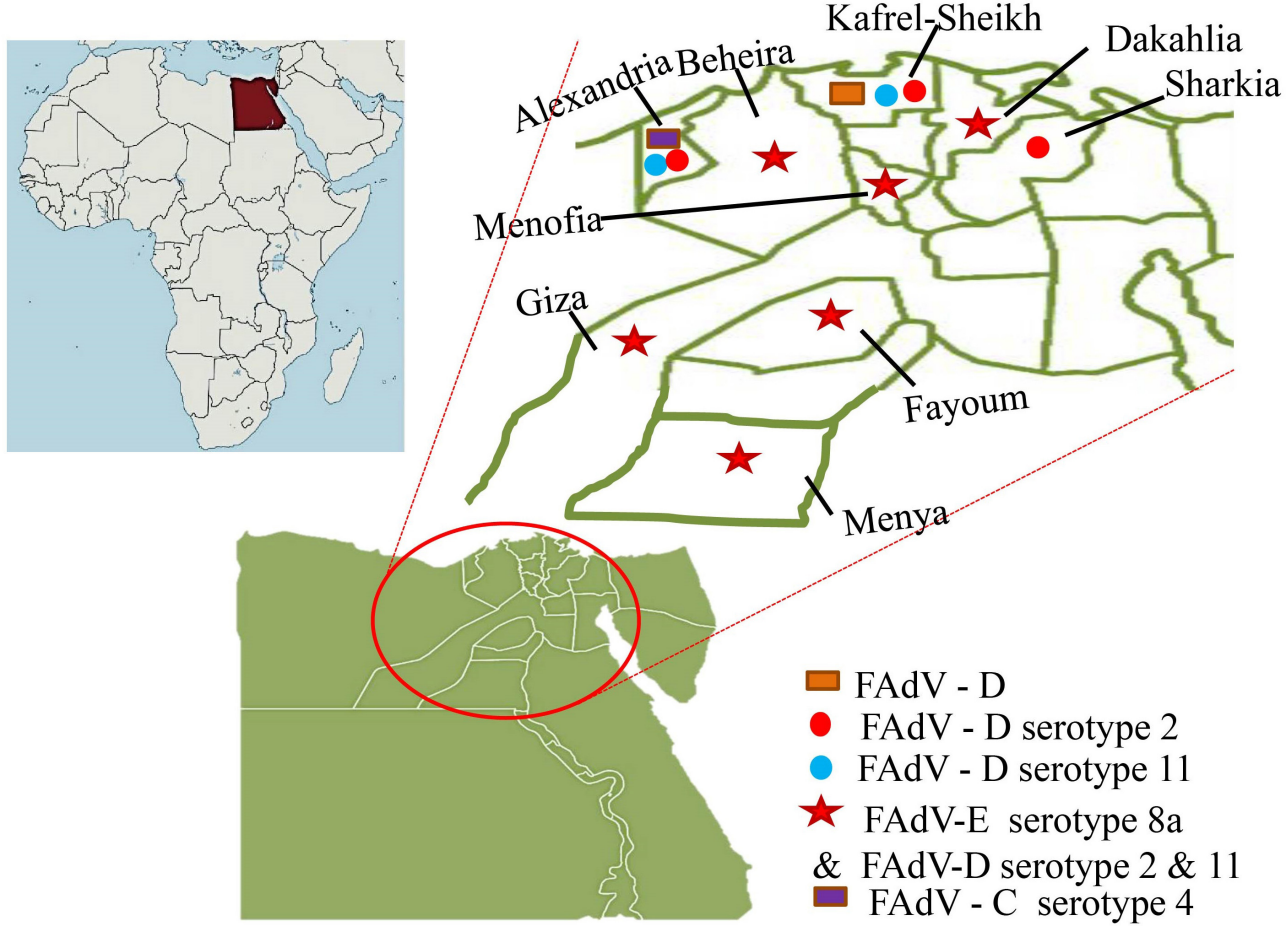
The recorded distribution of fowl adenoviruses (FAdV) in Egypt.

In terms of HHS, Sultan et al. (55) reported for the first time a pathogenic fowl aviadenovirus-4 (FAdV-4) in a Cobb-500 broiler flock aged 32 days in Alexandria governorate, Egypt in 2021. This flock had a high mortality rate (15%), depression, greenish diarrhea, a flabby heart with a pericardial sac filled with straw-colored fluid, a pale enlarged friable liver with hemorrhage, necrotic foci, and an edematous kidney. This FAdV-4 genotype was 98% identical to the Israeli strain IS/1905/2019. All of the recent studies were focused primarily on a region of northern Egypt (Figure 2).

As a result, additional research on the prevalence of HHS and other IBH serotypes should be conducted, including all Egyptian governorates and a wide range of poultry sectors. Furthermore, research into their pathogenicity, impact on immunity, interaction with other pathogens in the field, and association with severe economic losses in Egyptian poultry production became necessary.

## Etiology and classification

**Figure 3:**
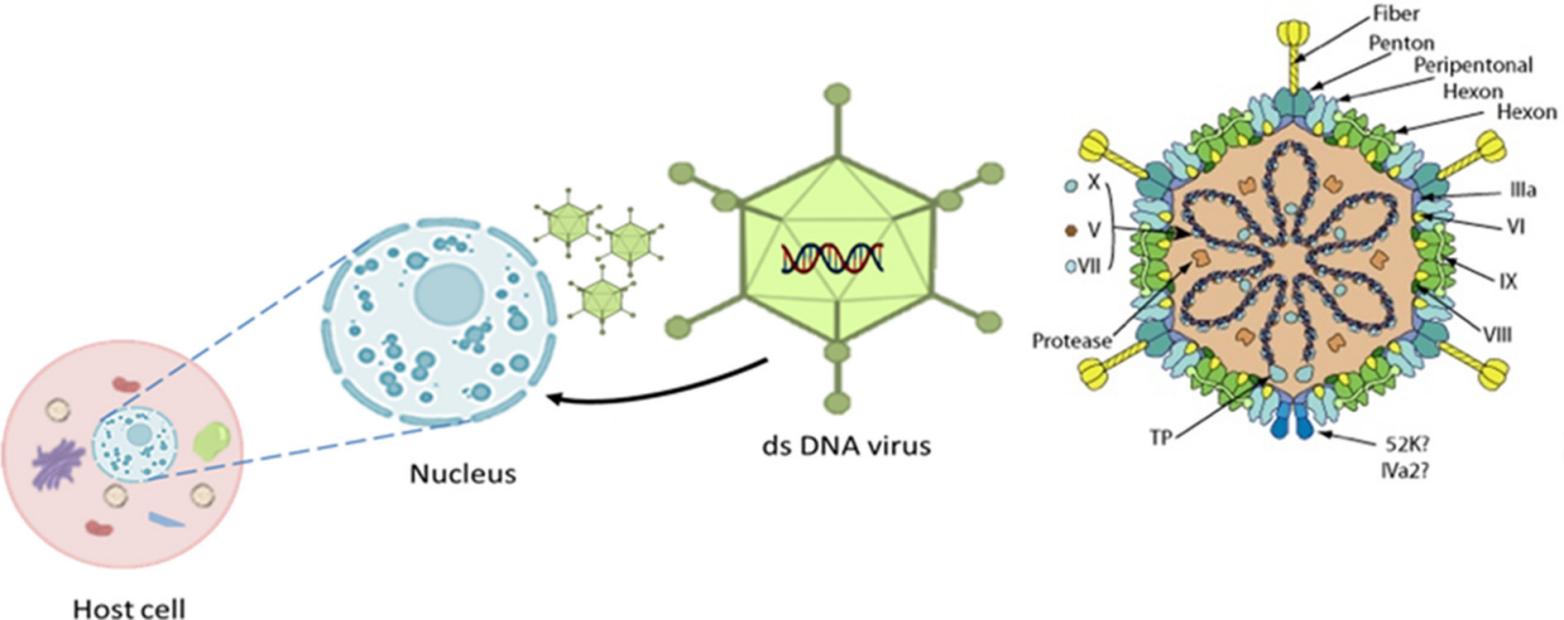
The 3D icosahedral structure of aviadenovirus with its pseudo-icosahedral capsid, genome, associated structural protein, and the fiber (252 capsomers, 240 hexons, and 12 penton bases carrying fiber protein projections). Replication takes place in the host cell nucleus. Modified after https://viralzone.expasy.org/4.

The causative agent, adenovirus, is a member of the family adenoviridae and consists of a non-enveloped capsid with pseudo-icosahedral symmetry (Figure 3) composed of 720 hexons arranged in 240 trimers and 12 vertex pentons each with two fiber proteins protruding to the surface that are transcribed from a single fiber gene except for FAdV-A and FAdV-C which have two genes (56). Its genome is composed of 35–36 kb of linear, monopartite, double-stranded DNA that encodes ~40 proteins. The DNA contains terminally redundant sequences with inverted terminal repeats and a terminal protein attached to each 5′ end of the ds-DNA (56–58).

Hexon protein has group and type-specific epitopes (59), and it is involved in the production of neutralizing antibodies, which could provide protection. Aside from neutralization, this protein is linked to hemagglutination, virus infectivity, and pathogenicity, making it a virulence factor (60). Sequencing of variable regions (right and left flank) of hexons, DNA polymerase genes, or the entire genome has recently been used not only to assign adenoviruses to species levels but also to further subdivide them into genotypes or serotypes (61–63). Based on fiber 2 phylogenetic analysis, Liu and his co-authors (64) distinguished pathogenic and non-pathogenic FAdV-4 strains inducing HHS from various geographical regions. It can also be used to investigate amino acid substitutions to study FAdV-4 strain genetic variation and molecular evolution (64).

**Figure 4:**
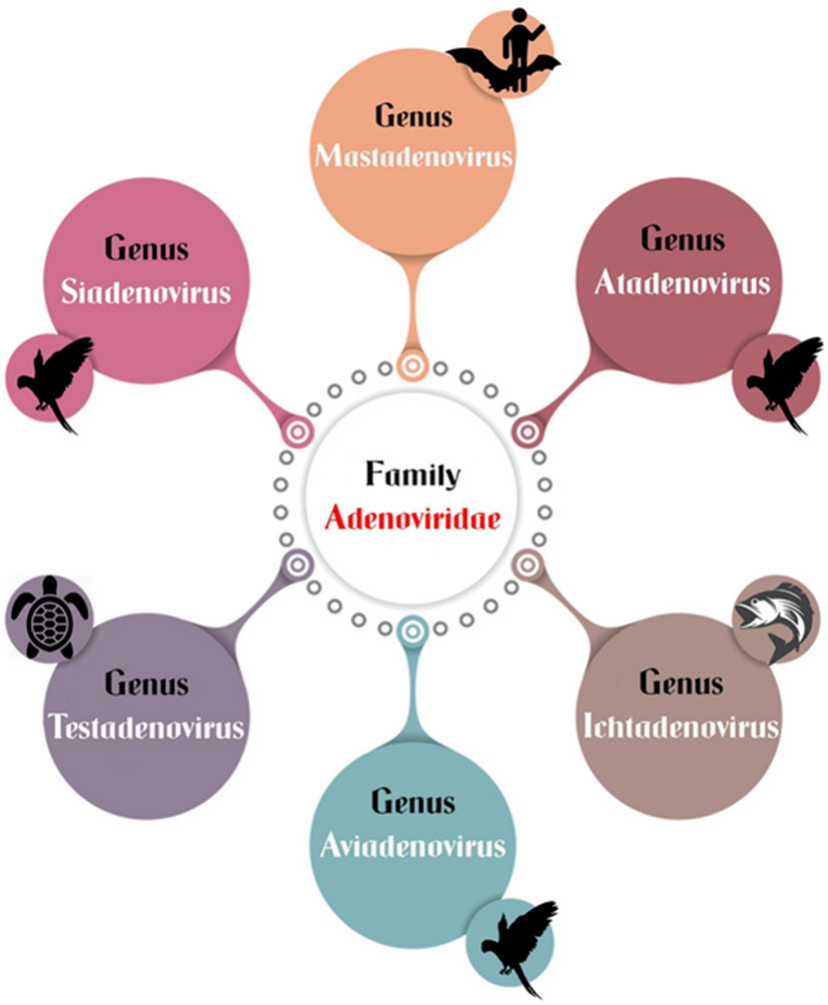
Different genera of adenoviridae.

Historically, avian adenoviruses were divided into three groups: group I (fowl adenovirus), group II (HE, MSD, and AAS), and group III (EDS_76_). The family adenoviridae has recently been divided into six confirmed genera: mastadenovirus, aviadenovirus, atadenovirus, siadenovirus, and ichtadenovirus, as well as the proposed testadenovirus of turtles and tortoises (Figure 4). Three of them can infect birds (aviadenovirus, siadenovirus, and atadenovirus), and viruses within each were classified into species, which can be further classified into serotypes (64). This classification was based on the most important criterion, phylogenetic distance, along with at least one of the following: cross-neutralization, genome organization, guanidine and cytosine (G+C) content, and host range (65). Within the aviadenovirus genus, FAdVs are classified into five different species (FAdV-A to FAdV-E) based on their molecular structure, and 12 serotypes (FAdV-1 to−8a and−8b to−11), based on cross-neutralization tests (Table 1).

FAdVs isolated from IBH cases are most commonly FAdV-D and FAdV-E. FAdV strains associated with HHS are FAdV-4 (FAdV-C) and are highly pathogenic to chickens (66). FAdV-1 (FAdV-A) has been isolated from the majority of gizzard erosion (65). Although individual adenoviruses are usually hosting species-specific, adenoviruses from multiple genera can infect a single animal species. FAdV-D (serotype 2) and –E (serotype 6) causing IBH outbreaks were molecularly identified in diseased broiler chickens and falcons, in Saudi Arabia, respectively and these viruses were distinct from falcon adenovirus species A (67). As a result, they reported that cross-species transmission between chickens and falcons was possible. This phenomenon will result in recombination events that will result in the emergence of a new adenovirus with new host affinity and pathogenicity characteristics.

Das et al. (68) discovered novel recombination of the fiber-2 gene from poicephalus adenovirus (PoAdV) affecting parrots to FAdV-C, the latter is causing the most recent outbreaks of HHS in broiler chicken flocks around the world (68). Furthermore, Marek et al. (69) concluded that possible recombination events within different hosts (domesticated, wild, and psittacine birds) may explain the frequent rapid updates of adenoviruses incidence in poultry in recent years, and that future results cannot be predicted (69).

## Epidemiology, pathogenicity, and clinical signs

In the middle of the twentieth century, embryonic eggs became useful for producing vaccines as well as for isolating filterable chemicals from various sources. It was occasionally found that such procedures could isolate a virus unrelated to the original material (10, 70). As a result, vertical transmission was recognized as one of the first important biological characteristics of chicken adenoviruses, particularly at the height of egg production.

Losses in healthy chicks without co-infections are possible with vertically transmitted FAdVs (71). On the other hand, they may go unnoticed for a while and then reactivate in young birds with immunosuppression (72, 73). FAdVs are also easily horizontally transferred *via* the oral-fecal route because of the high shedding titers in the feces (74). Later, experimental infections and epidemiological studies were conducted to determine the role of FAdVs in IBH (70). Table 2 shows the induced IBH and/or HHS in birds infected with different FAdV serotypes experimentally or naturally.

**Table 2:** Induced inclusion body hepatitis (IBH) and/or hepatitis-hydropericardium syndrome (HHS) upon experimental or natural infection of birds with different fowl adenoviruses (FAdVs) serotypes.

**Disease**	**Bird type**	**Age**	**Adenovirus serotype**	**Route of inoculation**	**Dose**	**Mortality rate**	**Hepatitis**	**Hydropericardium**	**References**
IBH	SPF chickens	35 days	11	IM	10^7.5^ TCID_50_	0%	+	-	(75)
	SPF chickens	1 day	11	Oral	Half ml of 10^6.5^ TCID_50_ ml^−1^	0%	+	-	(33)
	SPF chickens	11 days	8b	Oral	10^8.53^ TCID_50_	3.8%	+	-	(76)
				IM		40%	+	-	
	SPF chickens	3 days	11	Oral	0.1 ml of 10^4^ ELD_50_ 0.2 ml^−1^	2.5%	+	-	(77)
	SPF chickens	1 day	2, 8	IM	10^6^ PFU	80–100%	80–100%	33.3–80%	(78)
		3 weeks	2, 8	IM	10^7^ PFU	0%	0%	0%	
	Broiler chickens	1 day	8a	Oral	10^7^ TCID_50_ ml^−1^	0%	+	-	(54)
	SPF broilers	1 day	FAdV-D	Oral	10^7^ TCID_50_ ml^−1^	96%	+	N.E.	(79)
			FAdV-E			100%	+	N.E.	
	SPF layers	1 day	FAdV-D	Oral		8%	+	N.E.	
			FAdV-E			20%	+	N.E.	
HHS	SPF chickens	1 day	4	IM	10^6^ PFU	100%	100%	80%	(78)
		3 weeks	4	IM	10^7^ PFU	20%	20%	20%	
	SPF chickens	5 weeks	4	IM	10^6^ ELD_50_	100%	100%	100%	(80)
				Oral		76.9%	100%	100%	
	Meat duckling	25 days	4	S/C	10^7.5^ ELD_50_	23.3%	+	+	(81)
				Oral		16.7%	+	+	
	SPF chickens	10 days	4	IM	10^7.5^ ELD_50_	50%	+	+	(82)
				Oral		23.3%	+	+	
		20 days		IM		16.67%	+	+	
				Oral		16.67%	+	+	
	SPF chickens	3 weeks	4	IM	10^5^ TCID_50_	100%	+	+	(83)
	Backyard chickens	6 months- 2Years	4	Natural infection	-	27%	+	-	(28)

+, reported but without a %; N.E., Not Examined; SPF, specific pathogen free; IM, intramuscular; S/C, subcutaneous; PFU, plaque forming units; ELD_50_, lethal dose_50_; TCID_50_, median tissue culture infectious dose.

It was shown that the virus colonizes the intestinal epithelium at 12 h post-infection (hpi) and can be found in the blood as early as 24 hpi after oral inoculation of chickens (74). As early as 2 and 3 days after infection, the virus is detectable in the liver and pancreas, which are its target organs. The incubation period is during which there are no clinical symptoms or obvious abnormalities (84). The fast viraemia that results from the virus multiplication after the incubation period and causing pathological lesions in its target organs occurs at the same time as the disease's clinical manifestation (74, 79, 84). Beginning around 79% recovery in birds is marked by a lessening of clinical symptoms, cellular regeneration, and a decline in viral load in the affected organs (79, 84).

The virus, however, is persistently secreted in feces and dormant in the caecal tonsils. Twelve weeks after SPF hens were orally infected with an FAdV-1 strain when they were just 1 day old, the virus was still detectable in their intestines (85). Additionally, rather than being related to the pathogenicity of a particular strain, the quantity and duration of virus shedding in the feces coincide with the viral dose supplied (86, 87). Due to the prevalence of the viruses vs. sporadic outbreaks and variation between experimental experiments, the pathogenicity of FAdVs as a single infection (primary pathogen) has long been debated (40, 78, 88–91). However, the importance of FAdVs as primary pathogens is still debatable because various strains of the same serotype may have varying levels of pathogenicity (86, 92, 93). Recent research has shown that FAdVs have been found in both healthy and ill chickens (94, 95).

### IBH

Historically, IBH was known to occur as a secondary or concurrent pathogen (opportunistic agents), particularly in the presence of immunosuppressive agents such as IBD, CIAV, avian reticuloendotheliosis virus (ARV), avian leukosis virus (ALV), and mycotoxins (96–100). Nonetheless, it was observed in recent outbreaks as an independent primary pathogen in various parts of the world, causing hepatitis and sudden death of up to 30% of broiler chickens aged 3–5 weeks, which usually peaked at the 4th days post infection (dpi) and may terminate by the fifth dpi, but deaths may extend for another 2–3 weeks (4).

IBH has been documented in chickens as young as 7 and 10 days old, as well as in 1-day-old turkeys, even though it typically affects poultry between the ages of 3–5 weeks (29, 101–103). Before passing away, the sick birds may exhibit despondency, ruffled feathers, and decreased meal intake (104). IBH can impact breeder, broiler, and layer hens. The route of inoculation, virus dose, age of the bird, serotype or even strain pathogenicity, and bird type are just a few of the variables that are considered when determining the disease result during various *in vivo* experiments (78, 105–107).

To cause a clinical manifestation of IBH in chickens older than 1 week old, an invasive route of infection, such as intramuscular or intraperitoneal, is typically necessary (91). Up till now, no study has conclusively defined this phenomenon, and the mechanisms causing it are not entirely understood. IBH affects different breeds of hens, which has a significant impact on how the disease progresses. Cook used a variety of chicken breeds of SPF and non-SPF origin to study the effects of host age and the manner of inoculation on the results of FAdV infection. Upon intraperitoneal inoculation, he found that SPF Light Sussex hens were more vulnerable to infection than SPF Rhode Island red chicks, with fatality rates of 67 and 33%, respectively (86).

More recently, FAdV-D or -E oral infection of SPF broilers resulted in a mortality rate of 96–100% with severe clinical signs, compared to 8–20% mortality in SPF layers inoculated under the same conditions (79), which was consistent with the situation reported from the field and the higher susceptibility of broilers. Similar to this, 20% of SPF layers and 40% of SPF broilers died after subcutaneous FAdV-E strain infection (108).

Furthermore, there is an age-associated resistance to IBH (over 3–5 weeks). Dar et al. (30) observed that the infection susceptibility increases in SPF leghorn chicks at 1 day old and declines as they get older. In addition, FAdV strain or serotype affects the virus's pathogenicity. However, no conclusive molecular data exists to distinguish FAdVs according to their pathogenicity (30). Animal challenge and evaluation of morbidity, mortality, and the severity of the lesions are the most reliable ways to assess the pathogenicity of FAdVs (109). On the other hand, co-infection with other pathogens such as IBD, CIAV, ND, and *Escherichia coli*, as well as the bird's immune status, viral dose, and route of infection, can all influence the outcome of IBH (109).

Furthermore, no statistically significant correlation between flocks exposed to FAdV and co-infected with CIAV or IBDV was discovered in a recent investigation (110). On the other hand, reports from Australia, New Zealand, Canada, and Japan reported IBH epidemics without the presence of risk factors, supporting the idea that FAdVs are a primary pathogen (36, 40, 78, 88–90). All 12 FAdV serotypes have been connected to IBH epidemics (111). However, serotypes 2 and 11 of FAdV-D, as well as 8a and 8b of FAdV- Serotypes 9 and 3 of FAdV-D were found in South Korea (107), serotype 7 of FAdV-E was found in Iraq (42), Poland (112), and China (43). Other serotypes have also been reported to be included in IBH (100). FAdV-E was found to be the most prevalent in different geographical regions (4).

Mittal et al. (113) identified FAdV serotypes 2, 4, 5, 6, 7, 8, and 12 in broiler chickens affected with HHS or IBH in India. Furthermore, an outbreak can include at least two serotypes; due to the lack of serotypes cross-protection. For example, the predominant combinations indicating mixed infection in broiler chickens in India were FAdV-8 and FAdV-5, FAdV-8 and FAdV-7, FAdV-8 and FAdV-6, FAdV-8 and FAdV-12 (113). Therefore, an autogenous vaccine covering all serotypes involved in such outbreaks should be used.

### HHS or Angara Disease

In Pakistan, sudden and high mortality in broiler chickens aged 3–5 weeks was reported as a new disease (Angara Disease) in 1987, and it was later reported globally with a mortality rate of 20–80%. It is caused by the highly pathogenic FAdV-C serotype 4 virus and is characterized by an enlarged, friable and hemorrhagic liver with INIB, pulmonary edema, and nephritis (114).

All these symptoms are similar to IBH, but the main difference between the two diseases is the presence of a typical hydropericardium caused by HHS. In India, HHS was dubbed “Lychee” disease because the affected birds' hearts were surrounded by hydropericardium which resembled a peeled Indian lychee fruit (115). It was suggested that testing the virus pathogenicity for chickens older than 3 weeks is an easy way to differentiate between IBH and HHS (78). HHS outbreaks have also been reported in Kuwait, Iraq, Russia, India, Korea, Japan, North, and South America, and Egypt (55, 66, 115–119).

The FAdV causing HHS was identified as serotype 4 by a serum-neutralization test, PCR assay, and restriction enzyme analysis. Inoculating isolated FAdV−4 subcutaneously and orally in 28–day-old broilers, resulted in disease reproduction (120, 121). The virus's affiliation with avian adenovirus serotype 4 was established through sequence analysis of the variable region hexon gene. India and Pakistan isolates were 94–98% homologous (122).

According to phylogenetic research using the entire nucleotide sequences of the short fiber genes, the FAdV-4 strains from HHS in Japan, India, and Pakistan formed a distinct cluster from FAdV-4 strains not originating from HHS (123). The phylogeny of the hexon gene sequences of two FAdV isolates from Anand, Gujarat in India revealed that both viruses clustered with chicken adenovirus 12 (strain 380) and fowl adenovirus 11 (strain C2B) and formed a small branch of the upper group, indicating the establishment of a novel fowl adenovirus genotype (124).

Later, the HHS was formed, which highlighted the significance of FAdV infections in young chickens, especially broilers, and elevated awareness of FAdVs as primary causative agents (91). The signs of an ill bird include lethargy, huddling with ruffled feathers, appetite loss, and mucoid yellow droppings. Infected flocks' lower weight gain has led to a low feed conversion ratio (125, 126). In the later stages of the infection, the chicks were dull, sad, with ruffled feathers, and reluctant to move, collecting in corners, according to Asrani et al. (126). The birds continued to be active until they died. Birds with HHS had severe anemia and a considerable decline in all hematological values, according to clinical pathology characteristics (127).

Additionally, albumin levels were altered in serum, whereas b-globulin levels rose (128). When the osmotic pressure of colloidal plasma is decreased, fluid leaks into the pericardial sac. While serum levels of uric acid, potassium, calcium, and triglycerides were significantly greater, blood glucose and plasma protein levels were decreased. This could be because of fluid buildup in the pericardial sac and the abdomen (129). Normal birds have low serum enzyme activities for aspartate aminotransferase, alanine aminotransferase, and creatine phosphokinase, whereas HHS-infected birds have high serum enzyme activities (130, 131). All these changes suggested that the liver, kidney, and heart were all involved in HHS.

## Pathology: Gross and microscopic lesions

### Gross lesions

The major liver abnormalities in IBH are pale, friable, enlarged livers with minute white foci and, in certain cases, petechial or ecchymotic hemorrhages. Glomerulonephritis is commonly accompanied by swollen kidneys (27). In both spontaneous and artificial infections, atrophied bursa and thymus, aplastic bone marrow, and hepatitis have all been observed (4).

Gross lesions peaked 4–10 days following infection in SPF hens with ocular or oral illness (84, 87). Broilers are more vulnerable to greater mortality because of the severe metabolic imbalance and the substantial damage of the pancreas in addition to the liver (79). In HHS compared to IBH, there are more severe gross lesions in the liver and kidneys. Pathognomonic for HHS is hydropericardium, which is described as a buildup of clear, straw-colored fluid in the pericardial sac (78). Pulmonary edema, a larger, discolored liver, and enlarged kidneys with dilated tubules are typical abnormalities (118, 132, 133).

Some cases of spontaneous infections were recorded with petechial hemorrhages, focal necrosis in the liver, severe congestion, and hemorrhaging with myocardium fracture (23, 134–137). Ascites and widespread necrosis of the pancreas have been documented in HHS severe epidemics (138). Different FAdV serotypes causes HHS and IBH, and HHS has a substantially greater fatality rate than IBH does (1). The main pathognomonic lesion in HHS, the hydropericardium, has not been reported in IBH (78). Accordingly, abrupt high mortality in broiler hens aged 3–6 weeks with hydropericardium, nephritis, and hepatitis is what constitutes suspected field cases (125, 139, 140).

### Microscopic lesions

INIB in the liver is a hallmark of FAdV infection, which has also been previously documented, in both IBH and HHS (30, 84, 133). The pancreas, kidneys, gizzard, and small and large intestines all found to have INIB (1). They are primarily big, spherical, irregularly shaped, and basophilic with a clear, pale halo with virus particles in those intranuclear bodies.

Additionally, the hepatic parenchyma displayed congestion, hemorrhages, centrilobular or diffuse hepatocyte degeneration, cloudy swelling, fatty alterations, multifocal areas of coagulative necrosis, and sinusoidal space enlargement (141, 142). The presence of lymphocytes, plasma cells, macrophages, and heterophils can also be seen in multi-focal coagulative necrosis (141). Steer et al. (84) classified the disease into three stages based on the presence and severity of hepatic lesions: incubation (1–3 dpi), degeneration (4–7 dpi), and convalescence (14 dpi).

FAdV-8b and FAdV-11 were found in the liver, kidney, and gizzard of most birds during the degenerative stages, and remained in the gizzard until convalescence. The kidneys displayed subcapsular hemorrhages, hyperemia, denuded tubular epithelium, tubular epithelium degeneration, necrosis, and moderate interstitial lymphoplasmacytic nephritis (141, 142). Glomerulonephritis is characterized by an increase in the glomerular area and the average glomerular cell count, which was observed during a severe outbreak of IBH (27). Glomerulonephritis was the prominent renal pathology recorded in a broiler flock infected with IBH and complicated with citrinin mycotoxicosis (142).

Pancreatic necrosis is possible (4) and myocarditis as well as heart hemorrhages have been observed in chickens and ducks that died from HHS (23, 136, 143) with focal accumulation of mononuclear cells (141, 142). Furthermore, depletion of B and T cells in lymphoid organs was significantly induced after virulent FAdV-4 experimental infection in SPF birds leading to a significant immunosuppression (144, 145). Therefore, lymphocyte depletion in spleen, vascular congestion and hemorrhagic focal areas was noticed (141) with associated reticular endothelial cell hyperplasia (142).

Similarly, lymphocyte depletion with hemorrhages and follicular atrophy in the bursa of Fabricius can be seen (1). Moreover, congestion, hemorrhages, edema, and mononuclear cell infiltration were present in the lungs of chickens infected with HHS (141). Sharma et al. (146) and Ahamad et al. (147) suggested that the persistent intravascular pulmonary pressure may be the cause of the edema and injury to the pulmonary vessels.

## Immunosuppressive effect of aviadenovirus

HHS caused by aviadenovirus is a relatively new humoral and cellular immunosuppressive disorder (145, 148). Although layer and breeder flocks of < 20 weeks of age were affected as frequently as broiler chickens, the disease manifested in a milder form with a < 10% mortality rate (113). However, there have recently been reports in China of aviadenovirus serotype 4 strains evolving to be hypervirulent (having a deletion of ORF19) and causing recent emergent outbreaks in layers with a 50% mortality in the absence of other pathogen co-infection (80, 133, 149). Furthermore, in HHS outbreaks in China, high mortalities in commercial duck farms with pericardial effusion and stunted growth have occurred (81, 143).

After experimental FAdV4 infection of SPF birds, atrophy of the bursa of Fabricius follicles, lymphocyte loss with hemorrhages, and significant immunosuppression in the form of B and T cell depletion in lymphoid organs were detected (144, 145). Steer et al. (84) discovered FAdV-8b and FAdV-11 in the bursa and thymus of the majority of birds throughout the degenerative stage. The depletion of the mucosal immune system in the respiratory tract and the FAdV-induced degenerative alterations in the spleen and bursa, organs crucial for both cellular and humoral immune response, increased the risk of infection with other avian diseases (50). The virus was also discovered in the bursa and/or gizzard of some birds between 2 and 7 dpi, despite the FAdV-1 isolate being determined to be a pathogenic (50).

The effects on lymphatic organs in the thymus and bursa atrophy in the case of HHS and IBH, particularly caused by FAdV-4, FAdV-8b, and FAdV-11 as primary pathogens, may indicate that these strains are involved in immunosuppression; however, possible coinfections should be considered (23, 84, 136).

## Diagnosis of aviadenovirus

### Isolation and identification of aviadenoviruses

The preferred specimens for viral isolation are feces, kidney, pharynx, and afflicted organs (e.g., liver in IBH). For FAdV, liver, kidney, or hepatocellular cancer cell lines from chick embryos were infected with a 10% suspension of sample material. Although chicken cells can be used, it is better to use cells from the same species when trying to isolate aviadenoviruses from other avian species (24).

Most aviadenoviruses cannot be successfully isolated from embryonated eggs, although some isolates of FAdV have been shown to infect the yolk sac. An adenovirus isolate's identification can be verified using electron microscopy. Immunocytochemistry is a technique for the detection of adenoviruses that involves staining infected cells with an avian adenovirus antiserum tagged with a fluorescent dye. Virus neutralization testing using the isolate against common reference antisera to each known serotype are required to identify the serotype of an isolated virus (24).

### Molecular techniques

FAdVs were genetically divided into five distinct genotypes, A through E, which included all 12 serotypes (150). Since over two decades, FAdV has been detected using PCR. Primers are mostly based on the hexon gene, and the sequencing of its variable sections is used to classify viruses into species A through E and to determine genotypes within species (24, 56, 61, 151–154). High resolution melting curve analysis or pyrosequencing can also be used to differentiate viruses based on variation within loop one of the hexon gene (155, 156).

For a small number of strains, applying the hexon and fiber gene sequences for typing reveals some inconsistencies (61). The PCR can be extended beyond aviadenoviruses by creating primers of additional conserved areas, but without further subtyping of FAdV genotypes (24, 154, 157). In comparison to virus isolation, nested and real-time PCRs have been reported to increase sensitivity and can be employed for quantification (158, 159). FAdV have been isolated and found in birds without lesions using the cecal tonsils. The liver and pancreas are the two internal organs that were attacked (74, 160).

Recent studies of the whole genome sequences of both pathogenic and non-pathogenic FAdV-4 isolates revealed probable genomic changes related to virulence. The two most significant structural proteins, fiber 2 and hexon, as well as nucleic acid insertions and deletions in ORFs 19, 27, 48, and 19 A, are the main areas of variation (64). After the initial whole-genome sequencing projects and the adoption of hexon-based molecular typing (151, 161, 162), FAdV sequence data started to quickly increase in the early 2000's (4).

The molecular studies and sequencing of both the fiber and hexon proteins increase our understanding of how FAdV strains have evolved over time, which in turn helps us develop methods to stop disease outbreaks in poultry farms. The fiber and hexon proteins are essential for FAdV infectivity because they encode virulence factors. While the fiber-1 protein is crucial for viral replication and assembly independent of virulence potential as opposed to infectivity, and the fiber-2 protein is more crucial for FAdV pathogenicity than the hexon protein. In tissue tropism, the hexon protein is crucial (163).

*In situ* hybridization (ISH) has been shown to be able to identify viral DNA in tissue samples (24). If these procedures are not accessible, the presence of DNA-containing viruses can be inferred from the staining of infected cell monolayers or tissue slices as well as from the appearance of intranuclear basophilic inclusions. Finally, current developments in the diagnosis of diseases were documented (122, 158, 164–167).

### Serology

Hemagglutination inhibition is not applicable since hemagglutination is a distinguishing characteristic of FAdV1 strains, some of which hemagglutinate rat and/or sheep erythrocytes (168). The results of the ELISA, or indirect immunofluorescence assay, rely on the antigen employed to identify group-specific antibodies (24). Although the existence of numerous serotypes makes interpretation more difficult, ELISA can be used for this purpose. Type-specific antibodies are typically discovered using the serum-neutralization test. First detected in serum on day 14, virus-specific antibodies peaked at statistically significant levels at days 21, 28, 35, and 42 (137).

It is possible to discriminate between infected and vaccine-exposed mice using the recombinant non-structural proteins 33 and 100 K (169). The incomplete hexon protein has a high sensitivity for the detection of homologous antibodies (170, 171). The level of local immunity at mucosal surfaces is not indicated by the presence of humoral antibodies (171).

## Intervention strategies to control IBH and HHS

### Management procedures to prevent or minimize FAdV disease

Preventing aviadenovirus infection is primarily based on biosecurity practices. Strict managemental practices, as well as cleaning and disinfecting of premises and equipment; restriction of entry and/or personal protection of visitors and vaccination crews into poultry house, all play an important role in IBH and HHS prevention (1, 172).

The resistance of aviadenoviruses to inactivation by heat (up to 70°C) as well as high resistance to common disinfectants (particularly lipid solvents such as ether, chloroform, and phenol) (173) resembles a significant challenge, particularly in poultry houses with impervious floors and walls.

Application of glutaraldehyde and calcium hydroxide liquid combination under ambient temperature of 21°C during the downtime inside and outside the house was successful in preventing the persistence of gizzard erosion caused by FAdV-1 infection (174). On the other hand, it has been reported that the susceptibility of human adenovirus to disinfectants varies depending on the strain, and some strains are less susceptible to disinfectants like liposomal povidone-iodine, peracetic acid, and formaldehyde (175). It would be tough to get rid of aviadenoviruses once they have infected chicken farms. Overall, the viruses are regarded as latent across the country (11).

Yamaguchi et al. (11) isolated and identified serotypes (FAdV-A) causing gizzard erosion, and 5 (FAdV-B) as well as 8b (FAdV-E) from apparently healthy broiler and layer breeder chickens (1–20 weeks of age) in Japan. Similarly, Meng et al. (100) isolated FAdV serotype 7 from parental and offspring generations of chickens, in addition to co-infection with REV, ALV, and CIAV. Therefore, effective FAdV control begins at the primary breeder level with optimum disinfection and vaccination as two parallel lines that could prevent infection and, as a result, protect against vertical transmission. However, the horizontal spread is a significant issue that should not be overlooked, and it takes some effort to keep a commercial flock free of FAdV infection. Furthermore, controlling and/or eliminating immunosuppressive diseases such as IBD virus and CIAV is also critical in reducing FAdV disease because they increase the pathogenicity of FAdV (176).

### Vaccination

In recent years, outbreaks of IBH and HHS have resulted in higher mortality in birds as compared to early milder outbreaks, resulting in severe economic losses to the poultry sector. As a result, it is possible that the virus's virulence has changed, causing HHS as well as IBH, resulting in high mortality (177). As several evidences indicated the primary pathogenicity of adenoviruses, vaccination with certain genotypes/serotypes may be becomes more appealing. Many countries employ both live and inactivated vaccines to combat IBH and HHS. The FAdV serotypes 4 and 8 are most commonly used in commercial vaccines preparation (177). However, for disease prevention and control in endemic areas, it is recommended that autogenous inactivated vaccines prepared from the prevalent serotype of FAdV be administered. Primary breeders with strict biosecurity practices used autogenous inactivated vaccines to prevent vertical transmission and ensure the transfer of maternal immunity from breeding flocks to their progeny (177).

In Pakistan, broiler chicks are routinely immunized against HHS using an avian adenovirus of serotype 4, with apparent efficacy in containing the illness (4). Broilers are occasionally immunized in Pakistan at 10 days of age if their mothers lack serotype-specific adenovirus antibodies or if maternal antibody transmission is irregular because of poor vaccination practices, leading to a high proportion of unprotected birds. Historically, numerous trials have been developed to prepare inactivated vaccines driven from embryos and cell culture (178).

Ahmad et al. (179) demonstrated that the formalized vaccine of aqua base liver organ has a faster but lower immune response than oil base tissue culture vaccine; however, both vaccines are effective against HHS. According to Reddy et al. (180), the HHS vaccine was not only effective against HHS but also influenced immune responses against the ND virus. In addition, prophylactic vaccination of broilers aged 10–15 days with a formalin-inactivated liver homogenate vaccine provided excellent protection in the field in Pakistan (179, 181–183). However, many efficacies and safety factors such as batch–to–batch variation in viral antigen quantum, incomplete inactivation, and contamination by adventitious avian viruses have been overlooked in this successful type of autogenous vaccine, which are major disadvantages that may jeopardize biosecurity (184).

In India, only inactivated oil emulsion vaccinations are used to prevent HHS, but only in suspected epidemic situations (185). A few weeks after vaccination, Kataria et al. (186) found that an inactivated oil emulsified anti-IBH-HHS vaccine made from fowl adenovirus produced in cell culture gave protection. Similarly, Gupta et al. (187) has created an inactivated vaccine against the HHS virus using chicken embryo kidney cell culture. When challenged with virulent FAdV−4, the vaccine provided 100% protection in broiler chickens.

In the USA, autogenous inactivated vaccines are commonly used in primary breeder flocks where biosecurity measures are strictly enforced. Full protection against IBH was achieved in breeders up to the age of 50 weeks by immunizing a grandparent stock at 10 and 17 weeks of age with an inactivated vaccine containing isolates of species FAdVD and FAdVE (108). Using a polyphosphazene and avian beta-defensin 2 as an adjuvant induced an antibody response and upregulated certain cytokines in the spleen even after *in ovo* vaccination with a dead FAdV8b vaccine (188, 189).

Breeders and broilers are both immunized with inactivated vaccinations in other nations like Mexico and Peru. If the right vaccination is carried out, the progeny of breeders acquires the maternal antibodies, protecting them from both an infection in the field and a clinical form of the disease. A large proportion of unvaccinated birds died in the age range of birds under 10 days old, either because they did not get serotype-specific anti-adenovirus antibodies or because their transmission was irregular as a result of poor vaccination (190). One study in Chile found that vaccinating broiler breeders against both CIAV and FAdV-4 provided better protection for progenies against HHS than vaccination against either disease alone (191).

In Australia, breeders were given a live vaccine (FAdV8b) 1-3 times between the ages of 9 and 18 weeks by drinking water; yet, IBH outbreaks in broilers persist. A heterologous strain was infrequently identified from ill birds in addition to an FAdV similar to the vaccine, indicating a lack of cross-protection (84). Contrarily, an inactivated oil-emulsion FAdV-4 vaccine generated high levels of protection against different serotypes in both vaccinated/challenged SPF chickens and broilers descended from vaccinated breeders (192).

In Egypt, an inactivated FAdV-C serotype 4 vaccine has been commercially available and used for vaccination of breeder chickens since late 2020; however, the cross-protection of this vaccine with the isolated FAdVs D serotype 2 and 11 and E serotype 8 a,b remains unknown and requires additional research (data under investigation).

Inactivated vaccines, subunit vaccinations, and genetically modified vaccines have been the focus of recent research on FAdV-4 vaccines (193–196). However, neither low pathogenic nor non-pathogenic live vaccines have been the subject of in-depth research. Live FAdV-4 has been attenuated by passing virulent FAdV-4 strains through chicken embryos or QT35 cells (145, 197), however it is unknown whether these attenuated vaccines have any protective effects on the developing FAdV-4.

Zhang et al. (198) discovered that the hexon gene is the key gene responsible for FAdV-4's high pathogenicity and created a non-pathogenic chimeric virus rHN20 strain based on the newly discovered FAdV-4. The immunogenicity of artificially rescued rHN20 as a live vaccine was tested in chickens using various routes and doses (198). The live rHN20 vaccine induced high titers of neutralizing antibodies against FAdV-4 and fully protected immunized chickens against a lethal dose of FAdV-4 without any clinical symptoms or histopathological lesions in the liver, and significantly decreased viral load in immunized chicken tissues, leading to consideration of this live vaccine candidate for preventing HHS in the poultry industry (198).

Regarding the innovative recombinant vaccines that have recently been introduced into poultry production, several research studies have been conducted in order to obtain a recombinant vaccine for FAdVs-IBH and -HHS. The structural proteins fiber 2 or penton base of FAdV-4 expressed as recombinant proteins induced a high level of protection against HHS (199, 200), compared to only 40% protection with the 100 K non-structural protein (201). Furthermore, the VP2 gene of IBDV incorporated into FAdV serotype 10 genomes demonstrated efficient expression and protection of challenged birds (202).

Recent studies showed that FAdV-E field isolates from both homotypic (FAdV-8a) and heterotypic (-8b) serotypes causing IBH were resistant to the recombinant FAdV fiber protein, which was generated from an FAdV-8a strain. The liver viral load was significantly reduced after the birds had the homologous challenge, but they were not clinically protected from infection by heterologous serotypes. Additionally, fiber immunization promoted the proliferation of CD4^+^ T lymphocytes while moderating the CD8^+^ T cell response and prevented challenge-induced changes in systemic monocytes/macrophages and T cell subpopulations, suggesting that FAdV-E, recombinant fiber represents a vaccine candidate to only control the harmful effects of homotypic infection (203).

Additionally, recombinant viruses were created using unique genotype FAdV-4 strains in which the fiber-2 gene's N-terminus was either knocked off or fused to enhance green fluorescent protein in order to prevent pathogenicity in SPF hens. However, the primary cause for worry was the fact that *in vitro* recombinant virus titers were much lower than those of wild-type FAdV-4 (204). These advancements, which range from severe primary pathogens to non-virulent vectors, show the diversity of FAdV (204).

Recently, Wang et al. (75) detected for the first time fiber-1 which directly mediates the infection of FAdV-4 through its shaft and knob domains, through superinfection resistance assay, interfering assay, and serum neutralizing assay. The chicken coxsackie and adenovirus receptor (CAR) homology recently known as a cell receptor for fiber-1 was identified by Pan et al. (205). The efficient protection against the lethal challenge of FAdV-4 in chickens conferred by the vaccination of the knob domain containing fusion protein not only confirms the significant roles of the fiber-1 and its knob domain in mediating the infection of FAdV-4 but also highlight a promising application of knob domain-based subunit vaccines against FAdV-4 in the future (205).

De Luca et al. (206) created a unique chimeric combination epitope from two different FAdV serotypes, FAdV-4 and−11, and tested its effectiveness to shield hens from both HHS and IBH at the same time. In comparison to the corresponding challenge controls, the vaccinated/challenged birds showed fewer clinical signs, limited hepatomegaly, lower levels of AST, prevented the atrophy of HHS-affected lymphoid organs like the thymus and bursa of Fabricius, and significantly decreased viral loads in the target organs. By using CRISPR-Cas9 and homologous recombinant procedures, Lu et al. (207) created a novel recombinant FAdV-4 that expresses the fiber of FAdV-8b, known as FA4-F8b, which can effectively guard against both FAdV-4 and FAdV-8b challenge in 2-week-old SPF hens. Their work has laid a foundation for development of vaccines against multi serotypes of FAdV in the future (207).

To demonstrate their full potential, recent developments are required in the creation of efficient vaccines (172, 203, 208–216), novel therapeutics (217–220), and effective prevention and control strategies (172).

## Conclusion and future perspectives

IBH and HHS are currently considered emerging poultry diseases; however, the pathogenicity of most isolates is still debatable. The severity of infection in the field is considerably worsened by co-infections with other immunosuppressive viruses as CIAV, IBDV, ARV, ALV, and Marek's disease virus (MDV). The best approaches for identifying both diseases in birds are clinical diagnosis and molecular detection using PCR. Breeders need to follow tight biosecurity procedures, appropriate management methods, and immunization programs to control these diseases. As a result, preserving the expansion of chicken sector depends on expanding research into diseases linked to adenoviruses.

## Author contributions

All authors equally contributed to writing this review article. All authors reviewed and approved the final version of the manuscript.

## Conflict of interest

The authors declare that the research was conducted in the absence of any commercial or financial relationships that could be construed as a potential conflict of interest.

## Publisher's note

All claims expressed in this article are solely those of the authors and do not necessarily represent those of their affiliated organizations, or those of the publisher, the editors and the reviewers. Any product that may be evaluated in this article, or claim that may be made by its manufacturer, is not guaranteed or endorsed by the publisher.
