# Corrigendum: Epidemiology, pathology, prevention, and control strategies of inclusion body hepatitis and hepatitis-hydropericardium syndrome in poultry: A comprehensive review

**DOI:** 10.3389/fvets.2022.1075948

**Published:** 2022-11-22

**Authors:** Nahed A. El-Shall, Hatem S. Abd El-Hamid, Magdy F. Elkady, Hany F. Ellakany, Ahmed R. Elbestawy, Ahmed R. Gado, Amr M. Geneedy, Mohamed E. Hasan, Mariusz Jaremko, Samy Selim, Khaled A. El-Tarabily, Mohamed E. Abd El-Hack

**Affiliations:** ^1^Poultry and Fish Diseases Department, Faculty of Veterinary Medicine, Alexandria University, Alexandria, Egypt; ^2^Poultry and Fish Diseases Department, Faculty of Veterinary Medicine, Damanhour University, Damanhour, Egypt; ^3^Poultry Disease Department, Faculty of Veterinary Medicine, Beni-Suef University, Beni-Suef, Egypt; ^4^Bioinformatic Department, Genetic Engineering and Biotechnology Research Institute, University of Sadat City, El Sadat City, Egypt; ^5^Smart-Health Initiative and Red Sea Research Center, Division of Biological and Environmental Sciences and Engineering, King Abdullah University of Science and Technology, Thuwal, Saudi Arabia; ^6^Department of Clinical Laboratory Sciences, College of Applied Medical Sciences, Jouf University, Sakaka, Saudi Arabia; ^7^Department of Biology, College of Science, United Arab Emirates University, Al-Ain, United Arab Emirates; ^8^Khalifa Center for Genetic Engineering and Biotechnology, United Arab Emirates University, Al-Ain, United Arab Emirates; ^9^Harry Butler Institute, Murdoch University, Murdoch, WA, Australia; ^10^Poultry Department, Faculty of Agriculture, Zagazig University, Zagazig, Egypt

**Keywords:** aviadenovirus, diagnosis, disease transmission, epidemics, fowl adenoviruses, poultry diseases, vaccines

In the published article, there was an error in Figure 2 as published, figure was incomplete. The correct figure appears below.

**Figure 2:**
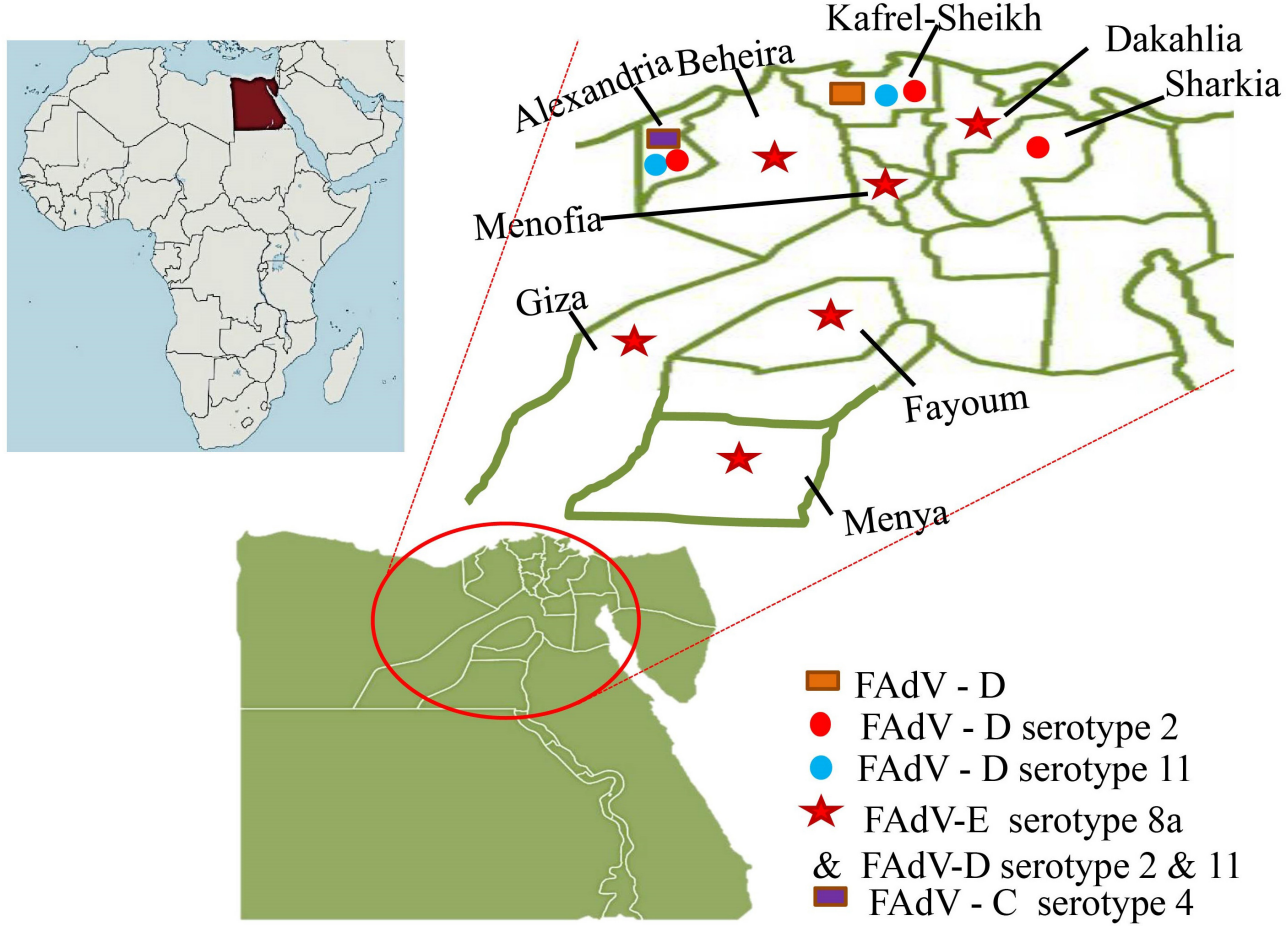
The recorded distribution of fowl adenoviruses (FAdV) in Egypt.

In the published article, there was an error in the caption of Figure 3 as published. The corrected caption appears below.

Figure 3. The 3D icosahedral structure of aviadenovirus with its pseudo-icosahedral capsid, genome, associated structural protein, and the fiber (252 capsomers, 240 hexons, and 12 penton bases carrying fiber protein projections). Replication takes place in the host cell nucleus. Modified after https://viralzone.expasy.org/4.

In the published article, there was a text error. A correction has been made to A brief history, paragraph number 5.

This sentence previously stated: “In addition, Bertran et al. (25) mentioned that the economic losses in Spain was caused by vaccination in broiler breeders due to increasing IBH cases.”

The corrected sentence appears below:

“In addition, Bertran et al. (25) mentioned that the dramatic increase in IBH cases caused major economic losses in the Spanish poultry industry, which prompted the use of vaccination in broiler breeders.”

The authors apologize for these errors and state that this does not change the scientific conclusions of the article in any way. The original article has been updated.

## Publisher's note

All claims expressed in this article are solely those of the authors and do not necessarily represent those of their affiliated organizations, or those of the publisher, the editors and the reviewers. Any product that may be evaluated in this article, or claim that may be made by its manufacturer, is not guaranteed or endorsed by the publisher.

